# Facilitating adherence to physical activity: exercise professionals' experiences of the National Exercise Referral Scheme in Wales. a qualitative study

**DOI:** 10.1186/1471-2458-11-935

**Published:** 2011-12-15

**Authors:** Graham F Moore, Laurence Moore, Simon Murphy

**Affiliations:** 1 Cardiff Institute of Society and Health, School of Social Sciences, Cardiff University, 1-3 Museum Place, Cardiff CF10 3BD, UK

## Abstract

**Background:**

Although implementers' experiences of exercise referral schemes (ERS) may provide valuable insights into how their reach and effectiveness might be improved, most qualitative research has included only views of patients. This paper explores exercise professionals' experiences of engaging diverse clinical populations in an ERS, and emergence of local practices to support uptake and adherence in the National Exercise Referral Scheme (NERS) in Wales.

**Methods:**

Thirty-eight exercise professionals involved in the delivery of NERS in 12 local health board (LHB) areas in Wales took part in a semi-structured telephone interview. Thematic analysis was conducted.

**Results:**

Professionals' accounts offered insights into how perceived needs and responses to NERS varied by patient characteristics. Adherence was described as more likely where the patient sought referral from a health professional rather than being advised to attend. Hence, professionals sometimes described a need for the referral process to identify patients for whom change was already internally motivated. In addition, mental health patients were seen as facing additional barriers, such as increased anxieties about the exercise environment. Professionals described their role as involving helping patients to overcome anxieties about the exercise environment, whilst providing education and interpersonal support to assist patients' confidence and motivation. However, some concerns were raised regarding the levels of support that the professional should offer whilst avoiding dependence. Patient-only group activities were described as supporting adherence by creating an empathic environment, social support and modelling. Furthermore, effectively fostering social support networks was identified as a key mechanism for reducing dependence and maintaining changes in the longer term.

**Conclusions:**

Whether ERS should identify motivated patients, or incorporate activities to support internalisation of motivation amongst less motivated patients deserves attention. As well as providing the knowledge to advise patients on how to exercise safely given their conditions, professionals' training should focus on providing the skills to meet the interpersonal support needs of patients, particularly where ERS are used as a means of improving mental health outcomes. The effectiveness of emerging activities, such as post-scheme maintenance classes, in fostering long-term social networks supportive of physical activity deserve attention.

**Trial registration:**

Current Controlled Trials: ISRCTN47680448

## Background

Physical activity is comparable to smoking and diet in terms of influence on chronic disease outcomes [1]. Hence, alongside population-wide physical activity promotion, interest is growing in interventions targeted towards populations whose conditions would benefit from physical activity [2]. One such approach, exercise referral schemes (ERS), has proliferated rapidly in recent years [3]. To date, randomised controlled trials and observational studies have provided limited evidence of long-term effects on physical activity [4,5]. However, despite substantial heterogeneity in their design [6], limited attention has been paid to understanding how the delivery of ERS might be improved [7,8]. Attending not only to whether ERS 'work', but also to 'what works, for whom and under what circumstances' [9] is crucial in attempting to improve their impacts.

The often limited long-term impacts of ERS have been attributed in part to limited uptake and adherence, with current evidence indicating that 1 in 3 referred patients do not attend a first appointment, while completion rates range from 12-52% [4,10]. Furthermore, recent observational studies have indicated that adherence is often patterned by patient characteristics. A number of studies for example indicate that mental health patients are less likely to complete ERS [7,11,12], that women are more likely to enter though less likely to complete [7,11,13] and that older patients are more likely to complete [11,14]. Socioeconomic patterning has varied across schemes. For example, uptake in one [15] was lower in deprived areas and among patients with lower education [16], while in another, participants were of lower SES than the population average, although those from the most deprived areas were least likely to participate [13]. Another reported higher referral volumes in deprived areas in London, with uptake and adherence equal between groups [14].

In attempting to improve the effectiveness of ERS, it is crucial to go beyond describing patterning in uptake and adherence, and towards understanding the reasons for this limited and often socially patterned reach. Gidlow and colleagues [8] have highlighted the need for qualitative research in order to explore these issues. Indeed, recent years have seen increasing numbers of largely qualitative studies exploring processes through which ERS might influence adherence and behavioural change. Perhaps the most common theoretical model guiding this literature is self-determination theory (SDT; [17]). SDT emphasises the need for interventions such as ERS to support three basic psychological needs; autonomy, competence and relatedness. Through supporting these needs, SDT argues that externally regulated changes, such as taking up exercise on instruction of a health professional, may over time become internally regulated. That is, performed for intrinsic enjoyment or because of associations with personally valued goals.

Studies have therefore focused upon the roles of components such as professional supervision and guidance in supporting these needs. Markland and Tobin [18] for example reported higher identified motivation (i.e. acting due to a sense of the behaviour as personally important) when patients perceived that professionals were supportive of autonomy and competence. An empathic, patient-centred approach was highlighted in one qualitative study as a welcome contrast to more authoritarian dealings with health professionals [19]. In another [20], the authors concluded that older women integrated exercise into their identities through development of a sense of mastery, as they were supported by the exercise professional. Indeed, access to an instructor who is both knowledgeable and provides effective interpersonal support has emerged as a key determinant of adherence in almost all qualitative studies [20-23]. Many early trials forming the basis for assessments of the efficacy of ERS however offered limited or unspecified professional support beyond consultations to discuss an exercise programme (e.g. [15,24,25]). Indeed, some qualitative studies have attributed poor adherence to limited continuity in professional support provision [26] or a perceived need for more interpersonal support than provided [27].

In addition to professional support, roles of collective exercise with patients in a similar position are emphasised throughout this evidence base as crucial in facilitating adherence [20-23,26,28,29]. In Markland and Tobin's aforementioned study for example, patients reported less external regulation when reporting higher levels of social assimilation into the exercise environment. Higher perceptions of personal relatedness (i.e. perceptions of supportive interpersonal relationships within the exercise environment) were associated with more internal regulation. Furthermore, Stathi and colleagues [28] conducted longitudinal interviews with older patients in a 12 month exercise programme, concluding that whilst support for knowledge, self-efficacy and competence was crucial in facilitating adherence, intrapersonal changes were more likely to lead to long-term behavioural change where accompanied by formation of long-term interpersonal networks. Structures for fostering social support networks have however been absent or not described within many ERS, with many simply offering discounted access to mainstream services. A minority of trialled schemes have focused on provision of group-based exercise opportunities [30,31], whilst few have explored how social networks are maintained in order to support ongoing activity. Indeed, qualitative studies identify disappointment amongst some patients at limited opportunities for social interaction within ERS, a desire for patient-only classes [23,29], or a sense of being 'dropped' after a short-term programme [31].

To date, evidence for the processes through which ERS support adherence and behavioural change has almost exclusively incorporated patient perspectives. Although professionals are well positioned to offer insights into issues such as which patients exhibit the most and least positive responses to such schemes, challenges and solutions in engaging patients in physical activity, and how practices are refined through experience to encourage uptake and adherence, fewer studies have explored views of implementers. The only studies incorporating views of exercise professionals have interviewed a small number alongside patients or referring professionals [28,32]. This paper presents analyses of interviews with exercise professionals within the National Exercise Referral Scheme (NERS) in Wales, exploring professionals' experiences of engaging diverse clinical populations in an ERS and emerging practices to support uptake and adherence. Interviews were conducted as part of the process evaluation of NERS, which was evaluated via a pragmatic randomised controlled trial, economic evaluation and mixed-methods process evaluation [33].

## Methods

### The NERS intervention

Discussions between evaluators and programme developers informed construction of a logic model which clarified key planned components of NERS (Table 1) and their intended functions in promoting behavioural change. The planned intervention began with referral to a local authority facility, for reasons including coronary heart disease risk factors (e.g. controlled diabetes or BMI > 28), anxiety or depression, musculoskeletal conditions and respiratory/pulmonary conditions. Alongside a health check to rule out contraindications, consultations were to be underpinned by motivational interviewing (MI) and goal-setting. Patients were then offered a discounted programme for 16-weeks, supervised by level 3 qualified exercise professionals, employed specifically to deliver the scheme. Patients were to be primarily offered group-based patient-only exercise opportunities. Although allowing local definition of exact form, the programme was to involve a variety of activities and class times, in order to appeal to a wide audience. Patients were to be encouraged to attend twice weekly, with access restricted to supervised sessions for at least 4 weeks. Professionals were to maintain dialogue with patients throughout the scheme and beyond, contacting those who dropped out before 4 weeks to encourage them back, whilst reviewing progress with attendees at 4 weeks and on scheme exit. On scheme exit, patients were to be signposted to alternative exercise opportunities. Eight month telephone contact was included to discuss progress since the scheme and discuss relapse prevention strategies, with a final health check and consultation at 12 months. However, implementation checks indicated that some components including MI (see [34]), goal-setting and patient follow-up protocols were not fully implemented, with the intervention centring around group-based, professionally supervised and discounted activity. NERS was introduced in 12 Local Health Board (LHB) areas who participated in a randomised trial, and subsequently to all remaining LHBs.

**Table 1:** NERS components as conceived and as delivered in practice

**Planned key components of NERS**	**NERS in practice**
Health professional referral to NERS	Health professional referral to NERS
Baseline consultation including	Baseline consultation including

• Health check and lifestyle assessment	• Health check and lifestyle assessment

• Motivational interviewing	

• Goal setting	

16 week exercise programme including	19 week (median) exercise programme including*

• £1 per class rate	• £1 per class rate

• Patient only group exercise classes as well as supervised gym use	• Patient only group exercise classes as well as supervised gym use

• Supervision by a level 3 qualified instructor	• Supervision by a level 3 qualified instructor

• Four week contact to discuss goals and experiences of the programme	• Contact to discuss goals and experiences of the programme.

• Contact of non-attendees at this stage to encourage back to programme	• Non-attendees typically not contacted.

Scheme exit consultation including	Scheme exit consultation including

• Repeat health check and lifestyle assessment	• Repeat health check and lifestyle assessment

• Discussion of goal progress	• Discussion of goal progress

• Signposting to exit routes	• Signposting to exit routes (including replacement of NERS discount with local discounts in most areas)

8 and 12 month follow up consultations to discuss progress since the scheme	8 and 12 month follow up consultations to discuss progress since the scheme (variable delivery)

	*indefinite access to NERS classes offered after scheme exit consultations by most professionals during the trial period

### Sampling and recruitment

Participants were exercise professionals appointed by the 12 LHBs participating in the NERS trial. Professionals' roles included delivering the service to patients, including one-to-one consultations and exercise classes, and assisting with collection of data for monitoring and evaluation purposes. Hence, professionals had contact with the evaluation team throughout the study period regarding its conduct and progress. Professionals on average, came into contact with approximately 10 new patients each month, having implemented NERS for 6-12 months at the time of interview. Areas differed in their histories of ERS, with most having previously offered varying local schemes. Furthermore, most retained professionals from these previous schemes whilst also employing new professionals. In order to represent this diversity of experience, all professionals in post at the time of interview (n = 41) were invited to take part in interviews lasting up to an hour regarding their experience of delivering NERS. Thirty-eight participated, with 1 missing 2 appointments while 2 did not reply. Three attempts were made to contact professionals before they were classed as non-responders.

### Data collection

Given the focus of the research on professionals' experiences of ERS delivery and its largely inductive nature, a qualitative approach using semi-structured interviews was adopted. Telephone interviews were chosen over face-to-face interviews for pragmatic reasons, given the geographical coverage of the scheme and cost of travelling to interviews. Information sheets were distributed to professionals and coordinators in the months before interviews took place. All interviewees provided informed consent for recording and use of anonymised quotations. Interviews commenced with a brief recap of the purpose and format of the interview and a reiteration of assurances of confidentiality, before being guided by a semi-structured interview schedule (see Table 2). The schedule was neither prescriptive nor exhaustive, using open-ended questions to probe topics of interest to the researcher, whilst allowing discussion of emerging issues of importance to the interviewee. Most lasted approximately 45 minutes, though where exceeding the hour initially advised, professionals were asked if they wished to end the interview. None chose to do so. The evaluation received approval from the Thames Valley Multi-centre Research Ethics Committee.

**Table 2:** Topic guide and example prompts/subtopics from interviews with exercise professionals

Topic	Example prompts
1. Role of the exercise professional	What does your role as an exercise professional on the scheme involve? What kinds of support and guidance do patients look for from you? How diverse is the type of support required by different patient groups?

2. Opinion of scheme and integration into context	What are your thoughts about: - the referral process? - the exercise facilities? - training provided? - the scheme as a whole? Is there anything about the area in which you work that has made it easier or more difficult to implement the scheme?

3. One-to-one consultations and motivational interviewing*	What do you see as the main purposes of your one-to-one consultations with clients? To what extent do you base your consultations on motivational interviewing principles (and in what ways)? Do you feel that the training received at the beginning of the scheme enabled you to do this (and how about after the refresher training course you recently received)?

4. Perceived changes and processes of change	Do you think that the scheme has been effective in changing patients' physical activity/psychological well-being? Do you think the scheme is more effective for some client groups than others? Do you think it should be targeted towards specific population subgroups? What is it about the scheme that you think helps clients to become more active? Do patients have any anxieties about the leisure centre environment (if so, who, how overcome)?

5. Motivation	How motivated, or ready to change, do you feel that clients are when they enter the programme? Are any patient groups more or less motivated on entry to the programme?

* included primarily to explore challenges in implementation of MI, as reported elsewhere [34]

### Analysis

Given the exploratory nature of the research, a predominantly inductive thematic approach to analysis was adopted [35]. Data were transcribed verbatim, with sound files encrypted and stored in a password protected folder. GM checked transcription accuracy, and initial generation of codes began during accuracy checks, focusing upon identifying talk regarding patterning in responses to the programme and how uptake and adherence to physical activity was supported. Each transcript was coded using QSR Nvivo. Codes generated from earlier transcripts were applied to subsequent transcripts. Earlier transcripts were revisited as new codes were identified. Coded data were then grouped into distinct themes and reviewed through rereading coded material and full transcripts. Keyword searches were also used in Nvivo to identify overlooked material in support of or deviating from the perspectives identified. The initial thematic framework was developed by GM. Draft analyses were circulated to all authors, and the framework refined and agreed in face-to-face meetings.

## Results

Themes and sub-themes from thematic analysis are presented in Table 3. Each is described in turn below, with anonymised quotations selected to illustrate a range of perspectives.

**Table 3:** Themes and sub-themes from thematic analysis of qualitative interviews

Theme	Subthemes
1 - Individual differences in needs and responses to NERS	1a - The referral process: motivating patients or identifying motivated patients 1b - Demographic patterning in responses to NERS

2 - Facilitating uptake, adherence and long term behavioural change	2a - Promoting uptake through overcoming initial anxieties 2b - Supporting confidence and motivation through education and interpersonal support 2c - Modifying the exercise environment to minimise anxieties throughout the programme 2d - Fostering social networks supportive of long-term change

### Individual differences in needs and responses to NERS

#### The referral process: motivating patients or identifying motivated patients

Whilst all patients entering NERS had done so following referral from a health professional, approximately half of professionals identified a distinction between patients who sought the programme, and those advised to take part by their health professional. In all such cases, health professional advice was seen as a weaker determinant of adherence than the patient's self-determined decision to seek help.

(8) Even if the doctor has told some clients that they need to go and do some exercise, that's still not enough of a culture shock for them, but the ones that decide, or saw the leaflet in the doctors and had to ask the doctor about it, generally they stick around.

Only one professional described a role for health professional referral as a motivator of change, suggesting the esteemed role of GP may have led patients to act on advice to change.

(5) If they go to their GP and their GP makes them aware of their behaviour, then they think 'oh god this is not my family nagging me now, this is somebody that's medically trained' and it makes them aware of their condition a little bit more.

High levels of drop out in some areas were attributed to failures to identify patients who were sufficiently motivated to benefit from the scheme. Hence, professionals sometimes described a need to focus attentions on patients for whom change was already internally motivated, rather than directing efforts towards motivating patients less ready to change.

(40) The drop out is I think a little bit too high at the moment. But the people that are really motivated coming in, they honestly, their lives have changed so much, it just makes it worthwhile and it is worth doing. If the right people are being referred in.

Most did not explicitly discuss how or at what stage motivation should be identified. One professional however, commented on roles for health professionals and implementers in ensuring that patients' understood the scheme prior to seeking their agreement to refer them through, ensuring that the scheme was offered only to patients interested in taking part.

(37) Quite often I think physios or any other health professional refer us in because they've, for want of a better word, run out of ideas. So they come to us these people, who haven't had the whole thing explained to them, so they're not sure really what's going on. I'm not sure if that means we need to promote it more in the community with more advertising to let people know it's here or whether that's for health professionals to work through.

#### Demographic patterning in responses to NERS

Following health professional referral, patients' responses to NERS were commonly seen as varying according to their conditions and demographic factors. In particular, professionals commented that whilst benefiting significantly where they did participate, engaging mental health patients in the scheme was often challenging.

(8) People with the mental health issues, if we get them along and keep them coming and keep them interested they see huge benefits but they tend to be the group that drop off.

This was attributed by many to a perceived lack of confidence and additional anxieties about assimilating into the exercise environment among mental health patients. Such anxieties were often also seen as particularly prevalent amongst older patients for whom the environment was more alien, and women or overweight patients, perceived as often particularly self conscious in the exercise environment.

(6) I think people with say like depression, they sort of, they kind of think people are talking about them and stuff like that - that's the sort of feeling I get - I've had one or two actually ask me if so and so was saying something - you know - just a little bit insecure that way. And I mean one or two of the older people, who are retired, they sort of feel that it's a young persons' sort of thing, going to the gym.

However, as most referrals were older women, some professionals commented that younger patients or men sometimes appeared to assimilate less easily into the patient-only classes, benefitting less from social aspects of participation.

(1) I suppose the youngsters that we get through are injuries or depression, do kind of drop off because they probably feel a little bit awkward because of the older people.

When asked whether there was anything about their area that made it harder or easier to implement NERS than it might be elsewhere, more than a third of professionals also identified perceived socioeconomic variations in responses to the scheme. Though one commented that patients in more deprived areas had appeared particularly grateful for the service, most others commented that engaging clients, in terms of both uptake and adherence, had been more difficult in deprived areas. This lower perceived engagement was attributed to factors such as a perceived lower tendency for poorer patients to place value on maintaining health, or a lack of buy in among GPs in more deprived areas.

(6) Its probably one of the hardest valleys to get the GPs to sort of buy into the scheme ... It's an ex-mining valley sort of thing, and it's very negative, it's like 50% unemployment. So they are kind of 'poor me' sort of thing, and they won't do anything to sort of progress themselves, if it doesn't involve say a pub or a restaurant, they're not interested.

Although NERS reduced cost barriers by offering classes at £1, some commented that patients were often not aware of these discounts, whilst others commented that long-term maintenance of attendance after expiry of the discount proved challenging for many.

(37) It's not the most, in terms financially affluent area. So obviously they struggle if anything, they seem to be able to cope with the pound cost for their sessions here, but then the progression afterwards is obviously quite awkward for them. Because once the pound stops if they're not willing to have the £15 for their gym membership, there's very little else to go to that's free.

### Facilitating uptake, adherence and long term behavioural change

#### Promoting uptake through overcoming initial anxieties

Professionals reported that the idea of entering NERS provoked anxiety for many patients, with initial anxieties perceived as stemming from worries about having confidence undermined by the presence of fitter exercisers, fears about assimilating into an unfamiliar social environment or fears of being expected to do exercises they weren't able to do.

(1) They are just worried about what people will think of them, they think the people there, everyone there is going to be fit, in their lycra and looking really smart but so that's the main thing, they just don't know, it's the fear of the unknown, they don't know what we are going to do with them

Hence, for some, development of strategies to overcome anxieties was seen as central to facilitating scheme uptake and adherence, sometimes beginning in the time between referral and entry to the exercise programme. One professional for example commented that during initial telephone contact, advertising the availability of patient-only classes in which fitter mainstream users would not be present had led to good responses, whereas another talked of sending out information packs about what the scheme would entail prior to scheme entry.

(22) They say yes it's quite daunting coming into the leisure centre for the first time, they're not too sure what they are going to be doing ...so we are trying to design a leaflet now which we are going to put out with the card itself saying exactly what they are required to do.

Initial consultations were often cited as an opportunity to reassure patients that professionals would serve as a familiar point of contact, as well as offering assurance that patients would not be expected to do anything that they were not confident about doing or which made them uncomfortable. Highlighting at this stage that the patient would be surrounded by others in the same position was seen as playing a substantial role in assuaging anxieties.

(39) They're very often afraid of the gym so we try and take away those barriers by being beside them in the gym for the first couple of weeks. We explain that we're going to be there and it's going to be a regular familiar face. They're quite reassured to know that whoever else is with us, are in the same position as they are.

#### Supporting confidence and motivation through education and interpersonal support

Patients were often described as lacking the knowledge of how to exercise safely given their medical conditions, with education crucial in allowing them to become independent exercisers without aggravating existing illnesses or injuries. However, whilst professionals varied in the emphasis they placed on educational or interpersonal support functions of their role, comments regarding a need for education were commonly inseparable from talk of the need to provide interpersonal support for confidence and motivation.

(5) Because of the training we've been through. Knowledge about conditions and what exercise then would suit them. Like I say, all the professionals here are supportive of their client's needs and understanding towards different problems that may occur, maybe anxiety, or confidence, or other problems with illnesses.

Indeed, some commented that instructional aspects became secondary to interpersonal support roles, given the vulnerabilities of the client group.

(19) My role is a motivator and mentor almost and um a support and someone that people can relate to and talk to openly about their situation so, I would say that's the first thing and then you are almost a fitness instructor second. Because the clients are quite vulnerable.

As described above, mental health patients were commonly described as facing particular difficulties assimilating into the exercise environment. Nevertheless, while some commented that this led to lower uptake or adherence, several others described particular successes engaging mental health patients. These professionals argued that additional barriers had been addressed through provision of additional interpersonal support or actively fostering interactions with other patients, with mental health patients then benefiting from improvements in confidence via exercise and socialisation.

(25) Just give them that little bit more support when they come in just chat to them a little bit more and make sure they work in pairs...I find that they are the ones most likely to stick at it more to be honest because they reap the benefits...the first couple of weeks are hard but they're the ones that stick at it

However, though typically speaking positively about the opportunities for training and development within NERS, several professionals identified a perceived need for further training to help them achieve similar successes in engaging these patients in the scheme.

(14) If they are depressed and you have the days you don't feel like coming, you are not going to come. You know again, the mood thing, their barriers as well are harder to break down. So a little bit more training in that area would be useful.

Some spoke of becoming valued components of patients' social networks as they supported them through the programme, with patients often seeking someone they could trust and with whom they could discuss issues affecting their wellbeing which were not always related to exercise.

(27) I had a client this week who I only said 'are you ok' and she started crying to me, so it was clearly nothing to do with the gym, but she felt that she could just talk to us and I suppose let off her emotions so. So yeah, clients look for us to be able to help them, but also I suppose be like a friend you know, be their support network.

However, while most appeared happy to offer this level of support, a minority expressed discomfort with the notion of becoming 'counsellors' to patients, with this seen as distracting from the main aim of promoting activity.

(34) You know listening, it's a good idea to listen but, you know when you've got your clients who want go into too much depth over things, and a bit too personal... it's not what you're here for, I don't think.

Hence, some commented on the need to balance potential benefits of providing the interpersonal support necessary to maintain motivation and confidence, against risks of patients coming to depend on an unsustainable level of support.

(14) Certainly with the older ones, there is a slight dependency trap with them, they do still like to come when you are there. It's quite hard sometimes to get them to exercise on their own.

Indeed, concerns about some patients' inability to maintain adherence without ongoing support had led to widespread tendencies for allowing some patients, particularly those perceived as being most vulnerable, to continue attending NERS classes after they had been officially exited from the programme.

(30) It's just a shame that we have to let them go at 16 weeks. But as I say, they've all got classes to go into and I wouldn't let anyone go who I thought was uncapable of being by themselves, which is why I have kept a few on, and they probably won't ever leave. But that's fine. If I didn't do that I wouldn't be safe in the knowledge that they'd be out by themselves.

#### Modifying the exercise environment to minimise anxieties throughout the programme

Whilst some described the aforementioned anxieties relating to the exercise environment as arising from preconceptions which dissipated once the professional accompanied patients and allowed them to see that their concerns were unfounded, anxieties were commonly seen as lasting to some degree throughout the programme. Hence, professionals commented on a need to structure classes in a manner which made patients feel at ease. While most areas limited group classes to patients, supervised gym sessions were most commonly held during public opening hours, with professionals therefore describing strategies such as arranging sessions during quiet times, to reduce the number of mainstream exercisers in the gym.

(7) We try to look at trying to get them in during quieter times when the age range is more mixed or more for their age range, as opposed to coming in at peak time when there are a lot of students here working at a lot higher intensity than they would be.

About a quarter of professionals served centre's operating a fully exclusive model, with separate gyms for referred patients, or the main gym closed to the public during NERS sessions; a model seen as highly beneficial in promoting programme adherence.

(3) It is an exclusive gym for that type of client, it tends to make it easier for the person to sort of integrate, and come in on board, whereas in the mainstream gym, they may have pre-conceived ideas about who's going to be in there, how many's going to be in there, what they are going to be doing, so that tends to sort of put them off really.

However, whilst perceived as beneficial in maintaining scheme adherence, some expressed concerns that allowing patients to entirely avoid mainstream exercise environments rather than providing a supported introduction, meant that patients continued to feel intimidated by these environments after the programme.

(1) The negative side is they don't want to go into the main gym, so it's kind of, they're wrapped in cotton wool because they've got their own which is great for the first sixteen weeks, but then we do have to try and push them on slightly, to integrate them in the main gym.

#### Fostering social networks supportive of long-term change

As described above, exercising with other patients was seen as helping to assuage patients' anxieties about the exercise environment, facilitating social assimilation. All professionals commented on a role for patients in supporting one another's adherence. Some spoke of the empathy patients offered to one another, having been referred for similar reasons and suffering similar limitations, removing the stigma associated with the process of struggling to overcome illness during classes.

(7) It's knowing that they are not on their own really the group tend to motivate themselves, and they will talk about what works for them, if they are having a bad day they will say they're having a bad day, they don't seem to be intimidated, they have got problems, it seems to be quite natural and they are not on their own.

Experiencing the scheme with other patients was seen as providing patients with realistic role models. New patients were able to observe others who had been in the programme longer than them doing things they weren't yet capable of, and could be encouraged to believe that they too were capable of achieving those improvements given time, rather than having their sense of competence thwarted by comparisons with healthier mainstream exercisers.

(40) They realise they're not the only person that's put weight on for example, that feels uncomfortable coming in. That there's several people that come into the gym and they say 'I've had this, I've had that' whereas not everybody wants to say why they're there but they're sort of 'look at me now' and you know 'I'm doing this and I'm doing that' and it sort of helps them to think that they can achieve their goals.

Whilst some saw interpersonal support as emerging spontaneously through group structures, describing a more passive role in facilitating interaction between patients, others saw explicit efforts to foster interaction during exercise classes as key to avoiding the aforementioned 'dependency trap', with emerging social support allowing professionals to gradually reduce support as patients progressed through the programme.

(29) I find that as an instructor they tend to follow you around 'will you be teaching the session'. 'Oh I'll miss that one then and wait for you to come back. You tend to get people like that ... I try and partner them up with a stronger person that I know. It's like putting them with someone in a similar situation that will also give them support.

Many commented that patients often continued to exercise with friends they had made after completing the scheme, although some remarked that loss of social aspects of patient classes were key reasons why some struggled to adhere to exercise in the long term.

(26) They love the people that they're with and they feel comfortable in that surroundings. And they're obviously feeling comfortable with myself. So you know what it's like, they don't like. A lot of people don't like change do they?

Hence, explicit efforts to foster emergence of social networks which lasted beyond the programme were seen by some as crucial in allowing patients to maintain adherence to exercise. Some talked of organising regular social events where current patients could meet one another, or others who had been through the scheme, or of strategies such as exiting patients from the scheme in clusters, and filtering patients into maintenance classes together.

(31) I never finish one on their own, even if it means that I keep them after assessing them another couple of weeks until someone else is exiting the scheme. I always try and buddy them...because we're feeding into maintenance classes, we find that a lot easier because there's the ones who've come off the scheme maybe a month before them there and then you've got maybe four more going in, and so they're really just the same group but at a different timeslot.

## Discussion

The present study offers a number of insights into how and for whom a national ERS was seen by implementers as facilitating adherence and behavioural change, as well as the emergence of local strategies for meeting patient needs within a national scheme. Firstly, in talking about the referral process, there was debate as to whether this should seek to motivate patients, or start from the assumption that patients will only adhere where change is already internally motivated. Markland and Tobin [18] comment that motivation is inherently external on scheme entry, given that entry is based on recommendation from an authority figure. However, contrary to this view, professionals commented that rather than acting on instruction, many patients had sought referral from their health professional, with the process of fully internalising exercise motivations perceived as more likely where motivations were already somewhat internalised prior to referral.

Some studies have focused upon assessing motivation for change prior to offering primary care based physical activity schemes [36,37]. This is however a controversial approach, in terms of the ethics of offering a scheme based upon a subjective judgment of motivation [38]. Rather than a fixed trait, motivation is largely a fluctuating product of social interaction [39], and expressions of ambivalence may reflect the manner in which the advice or offer of referral is presented to the patient. Interpreting resistance as a sign that a patient is unwilling to change may lead to the scheme being withheld from patients who would benefit. It may be that with future integration of evidence-based motivational communication strategies such as motivational interviewing [34,39] and effective goal setting processes, as recommended by Department of Health Quality Assurance Frameworks [6], the scheme may better engage patients who are initially more ambivalent about change.

In addition to the role of baseline motivation, professionals discussed perceived linkages between patients' responses to NERS and the conditions with which they were referred. Professionals commonly described greater barriers to uptake and adherence amongst mental health patients, including limited confidence and additional anxieties assimilating into the exercise environment. Demographic variations were also reported, with older patients and women described as exhibiting additional anxieties on entry to the scheme, though sometimes as finding it easier to assimilate and develop a sense of relatedness to others, due to the fact that most patients within NERS classes were also older females. Hence, the explicitly group-based structures may have gone some way towards offsetting tendencies for lower adherence amongst female participants observed in some ERS [7], though may have enhanced tendencies for higher adherence in older patients. Many also commented that the scheme was better received in more affluent areas, with lower engagement in poorer areas attributed to factors including lower motivation, limited financial resources and limited buy in among GPs. Findings on socioeconomic patterning in uptake and adherence of ERS are at present equivocal [13,14] and objective examination of such patterning is a priority for future analysis.

The roles of the professional in encouraging patients to take up the scheme were described as including development of effective strategies to assuage anxieties about entering the programme, consistent with reports of patients in previous qualitative studies regarding the intimidating nature of leisure centre environments [23]. Some identified initial contact about joining the scheme or initial consultations as opportunities to reassure patients that they would work alongside patients, and that they would not be pushed to do anything they were uncomfortable with. Hence, consistent with SDT [17], framing the programme in a manner which was autonomy promotive rather than controlling and did not threaten to undermine patients' sense of competence, was crucial in facilitating uptake.

Once patients attended, guidance on exercising within the limits of their conditions was described as crucial in providing patients with the skills to become autonomous exercisers without risking aggravating health problems. However, consistent with the dual roles of educator and provider of interpersonal support described in previous qualitative studies [19], such talk was commonly inseparable from discussion of the need to provide interpersonal support for confidence and motivation. Indeed, instruction was sometimes seen as secondary to mentoring roles given the perceived vulnerabilities of the client group.

Although as described above, professionals often identified lower adherence and greater anxieties relating to the exercise environment amongst mental health patients, some commented that where provided with sufficient interpersonal support, mental health patients benefitted substantially from the programme. Hence, the degree of opportunity for social interaction within NERS may have gone some way towards overcoming the often reported tendency for lower adherence among mental health patients [7,11]. However, variable reports regarding mental health patients' adherence perhaps reflected variable competence in supporting the needs of these patients. Indeed, some expressed a need for further training in dealing with mental health patients, or described discomfort with 'counselling' roles, with concerns expressed regarding the need to balance provision of sufficient interpersonal support to maintain motivation and confidence against risks of dependence upon unsustainable levels of support.

Consistent with research demonstrating the importance of social assimilation into exercise environments in enhancing patients' motivations for exercise [19,23,28], professionals placed substantial emphasis upon roles of other exercisers in supporting or discouraging adherence. Fitter mainstream exercisers were seen as playing a negative role, through providing unrealistic exemplars, undermining internal motivation through thwarting patients' need to feel competent. Conversely, an empathic social environment where it was normal to struggle with illness, and where patients could compare themselves against realistic role models who had progressed after attending the scheme for a while, was seen as helping patients feel that they could become competent, independent exercisers.

However, consistent with conclusions of a recent qualitative study that intrapersonal change are more likely to produce long-term change where accompanied by emergence of long term social support networks [28], professionals commonly discussed the contingency of ongoing exercise on the maintenance of networks developed during the scheme. Emerging strategies to lessen dependence on programme structures involved fostering social support networks through filtering completers into maintenance classes or exiting patients in clusters, with emerging networks taking over the role of the programme in motivating adherence to physical activity. Nevertheless, a tendency emerged in many areas for patients to be offered indefinite access to NERS classes, due to perceptions that continued activity was often contingent on ongoing support; a level of provision likely to become unsustainable as the scheme progressed.

Though strengths of the present study include a high response rate, with views of professionals in all areas delivering the scheme during the trial represented, the position of both the researcher and participants need to be considered. As typical of evaluative research, the interaction between an evaluator, linked to a trial of a scheme whose future hinged on positive findings, and professionals whose livelihood depend on its continuation, may have produced an understanding which portrayed the scheme in an excessively positive light. Hence, future research will focus upon the consistency of the trends perceived by professionals with findings from quantitative analysis of adherence and the extent to which psychosocial change processes were triggered by the programme, as well as perspectives of patients on the issues described in this paper.

## Conclusions

Whether the referral process should seek to direct resources toward motivated patients, or whether it is feasible to integrate activities such as motivational interviewing in order to enhance effectiveness for more ambivalent patients deserves consideration. Efforts to promote uptake among referred patients should emphasise aspects of the service which promote autonomy and which do not threaten to undermine patients' feelings of competence, thus assuaging anxieties regarding entry to the scheme. ERS professionals' training should emphasise providing professionals both with the skills to fulfil instructional roles, and the interpersonal skills to engage patients in physical activity. This appears particularly critical where dealing with patients likely to face additional anxieties assimilating into exercise environments, such as mental health patients. However, there is also a need to understand how patients' transition from the scheme ought to be best supported to avoid change becoming contingent on the support of the programme. Though some individuals may access ERS largely for the advice of an instructor without the need for wider social support, for many patients, provision of explicit opportunities for social interaction through patient-only group classes offers a means of lessening anxieties and assisting social assimilation into the exercise environment. This context potentially provides realistic role models to support patients' sense of competence, and opportunities to develop social networks supportive of longer term change. The effectiveness of emerging strategies to foster social networks to support the transition to autonomous activity, such as filtering patients into maintenance classes, or exiting patients in clusters, deserves close attention.

## Competing interests

The authors declare that they have no competing interests.

## Authors' contributions

GM led the development and implementation of the process evaluation under the supervision of SM and LM, conducted analyses and drafted the paper. SM managed the overall evaluation. All authors commented on drafts and approved the final manuscript.

## Pre-publication history

The pre-publication history for this paper can be accessed here:

http://www.biomedcentral.com/1471-2458/11/935/prepub
